# Supplementary material for: Profiling the Oral Microbiome and Plasma Biochemistry of Obese Hyperglycemic Subjects in Qatar
Source: Microorganisms. 2019 Dec 3;7(12):645. doi: 10.3390/microorganisms7120645 (PMC6955820; doi:10.3390/microorganisms7120645)
Supplement: Supplementary file 1 [file microorganisms-07-00645-s001.pdf]

Profiling the oral microbiome and plasma biochemistry of obese hyperglycemic subjects in Qatar

Muhammad U Sohail[1*], Mohamed A Elrayess[1], AA Al Thani[1,2], M Al-Asmakh[1,2], Hadi M Yassine[1,2*]

[1]Biomedical Research Center, Qatar University, 2713, Doha, Qatar

[2]Department of Biomedical Sciences, College of Health Sciences, QU Health, Qatar University, 2713, Doha, Qatar

Table S1. Data provenance*

Parameters	Grading					
	1	2	3	4	5	6
**Daily activity**	Sitting most of the time	Standing most of the time	Walking most of the time	Sitting, standing, and walking in equal amounts	Other work with moderate physical activity (includes moving or lifting objects of moderate weight)	Physically heavy work (includes moving or lifting heavy objects or activities)
**Breast feeding**	Yes	No				
**Weight gain during last 1 year**	No, weigh approximately the same	Yes, gained weight	Yes, lost weight			
**Oral Denture**	Yes	No				
**General Health**	Excellent	Good	Fair	Poor		
**Smoking**	No, have never smoked	No, just have tried once or twice	No, stopped smoking	Yes, only occasionally	Yes, on most or all days	
**Fast food**	Never or rarely	1-3 times per month	1-3 times per week	4-6 times a week	Once per day	2 or more times per day
**Processed Cheese**	Never or rarely	1-3 times per month	1-3 times per week	4-6 times a week	Once per day	2 or more times per day
**Hard cheese**	Never or rarely	1-3 times per month	1-3 times per week	4-6 times a week	Once per day	2 or more times per day
**Butter**	Never or rarely	1-3 times per month	1-3 times per week	4-6 times a week	Once per day	2 or more times per day

**Milk type**	Whole fat milk	Reduced fat milk	Fat free milk	All types of milk equally	Other; eg Soya, almond etc	I don't know
**Meat shawarma**	Never or rarely	1-3 times per month	1-3 times per week	4-6 times a week	Once per day	2 or more times per day
**Chicken shawarma**	Never or rarely	1-3 times per month	1-3 times per week	4-6 times a week	Once per day	2 or more times per day
**Falafel wrap**	Never or rarely	1-3 times per month	1-3 times per week	4-6 times a week	Once per day	2 or more times per day
**Samosa**	Never or rarely	1-3 times per month	1-3 times per week	4-6 times a week	Once per day	2 or more times per day
**Other middle-eastern style fast foods**	Never or rarely	1-3 times per month	1-3 times per week	4-6 times a week	Once per day	2 or more times per day
**French fries**	Never or rarely	1-3 times per month	1-3 times per week	4-6 times a week	Once per day	2 or more times per day
**Potato chips**	Never or rarely	1-3 times per month	1-3 times per week	4-6 times a week	Once per day	2 or more times per day
**Burgers, hotdogs**	Never or rarely	1-3 times per month	1-3 times per week	4-6 times a week	Once per day	2 or more times per day
**Pizza**	Never or rarely	1-3 times per month	1-3 times per week	4-6 times a week	Once per day	2 or more times per day
**Fried chicken, e.g. wings, nuggets**	Never or rarely	1-3 times per month	1-3 times per week	4-6 times a week	Once per day	2 or more times per day

*The demographic data was collected as per Qatar Biobank designed questionnaire. For statistical analysis the data were categorically arranged from 1 to 6 where 1 is lowest or negative value while 6 is highest possible value.

Table S2. Dietary habits of the study population. Consumption of dairy products

Group Statistics					
	group	N	Mean	Std. Deviation	P-Value
Milk	Obese	35	3.03	1.65	0.3
	Control	32	2.53	1.34	
Milk Cereals	Obese	35	2.11	1.47	0.1
	Control	35	1.57	0.78	
Flavored milk	Obese	36	1.78	1.12	0.7
	Control	34	1.59	0.92	
Laban	Obese	32	2.69	1.18	0.27
	Control	34	2.65	1.28	
Yoghurt	Obese	28	2.54	1.20	0.33
	Control	31	2.71	1.42	
Labneh	Obese	36	2.33	0.89	0.32
	Control	35	1.97	1.01	
Cheese	Obese	33	2.24	0.87	
	Control	32	2.13	1.24	0.06
Processed cheese	Obese	36	1.72	1.00	
	Control	36	1.86	1.25	0.34
Hard chees	Obese	36	2.00	0.99	
	Control	35	1.71	0.79	0.84
Butter	Obese	36	1.67	0.86	
	Control	36	1.86	1.20	0.17

Table S3. Dietary habits of the study population. Consumption of fast food

Group Statistics					
	group	N	Mean	Std. Deviation	P-Value
Meat shawarma	Obese	36	2.03	0.88	0.72
	Control	36	1.67	0.63	
Chicken shawarma	Obese	36	2.31	0.82	0.82
	Control	36	1.83	0.81	
Falafel wrap	Obese	36	1.92	0.69	0.2
	Control	36	1.67	0.72	
Samosa	Obese	36	1.69	0.62	0.52
	Control	36	1.58	0.65	
Other Middle East style fast foods	Obese	36	1.75	0.77	0.47
	Control	36	1.64	0.93	
French fries	Obese	36	2.25	0.94	0.56
	Control	36	2.11	0.95	
Potato chips	Obese	36	2.25	1.00	0.73
	Control	36	1.92	0.97	
Burgers hotdogs	Obese	36	2.33	0.93	0.61
	Control	36	1.97	1.03	
Pizza	Obese	36	2.22	0.87	0.47
	Control	36	2.06	0.89	
Fried chicken	Obese	36	2.22	0.90	0.3
	Control	36	1.89	1.09	

Table S4. Oral health of the participants

Oral Health	*No of participants responded	No of positive cases		Chi-square P value
		Obese	Control	
Mouth ulcers	43	1	2	0.79
Painful gums	42	0	2	0.91
Bleeding gums	47	4	3	8.80E-20
Loose teeth	43	2	1	7.74E-20
Toothache	62	12	10	8.06E-20
Oral denture implant	73	9	10	0.73

*Number of the participants responded yes or no to the oral health questionnaire. Chi-square test was applied on the oral health positive cases only.

Table S5. List of instruments and reagent kits used for serum biochemistry analysis

#	Test Name	Instrument Name	Reagent Kit used
1	ALT (GPT)	HG Architect C 8000	ALTL, 20764957 322, Cobas, Roche
2	AST (GOT)	ON RR Cobas 6000	ASTL, 20764949 322, Cobas, Roche
3	Alkaline Phosphatase	HG Architect C 8000	ALP, 10816388 216, Cobas Roche
4	C-Peptide	HG Architect i20	Elecsys C-Peptide, 03184897 190, Cobas, Roche
5	Cholesterol Total	ON RR Cobas 6000	cobas Lipid Panel, 06380115119, Cobas, Roche
6	Creatinine	HG Architect C 8000	CREP2, 05401470 190, Cobas, Roche
7	Estradiol	AW Unicel DxI 60	Elecsys Estradiol lll kit, 06656021 119, Cobas, Roche
8	Free Thyroxine	COBAS 801	Elecsys FT4 lll, 07976836 190, Cobas, Roche
9	Free Triiodothyronine	HG Architect i20	FT3 III, 06437206 190, Cobas, Roche
10	Glucose	HG Architect C 8000	GLUC3, 05168791 190, Cobas, Roche
11	HDL-Cholesterol	ON RR Cobas 6000	Cobas Lipid Panel, 06380115119, Cobas, Roche
12	Homocysteine	HG Architect i20	Homocysteine, 1L71/ABRL004/R4, Architect, Abbott
13	Insulin	HG Architect i20	Elecsys Insulin kit, 12017547 122, Cobas, Roche
14	LDL-Cholesterol Calc	ON RR Cobas 6000	Cobas Lipid Panel, 06380115119, Cobas, Roche
15	SexHormone Binding Globulin	HG Architect i20	Elecsys SHBG kit, Cobas, Roche
16	Testosterone Total	HG Architect i20	Elecsys Testosterone II, 05200067 190, Cobas, Roche
17	Thyroid Stimulating Hormone	COBAS 801	Elecsys FT3 lll, 06437206 190, Cobas
18	Triglyceride	ON RR Cobas 6000	cobas Lipid Panel, 06380115119, Cobas, Roche

Complete list of instruments used at Hamad Medical Cooperation Diagnostic Laboratory for analysis of the plasma biochemistry.
